# Differential expression of exosomal miRNAs between breast cancer patients with and without recurrence

**DOI:** 10.18632/oncotarget.19482

**Published:** 2017-07-22

**Authors:** Aiko Sueta, Yutaka Yamamoto, Mai Tomiguchi, Takashi Takeshita, Mutsuko Yamamoto-Ibusuki, Hirotaka Iwase

**Affiliations:** ^1^ Department of Breast and Endocrine Surgery, Kumamoto University Graduate School of Medical Sciences, Kumamoto 860-8556, Japan; ^2^ Department of Molecular-Targeting Therapy for Breast Cancer, Kumamoto University Hospital, Kumamoto 860-8556, Japan

**Keywords:** exosome, microRNA, breast cancer, circulating biomarker, prognostic factor

## Abstract

**Background:**

Exosomal microRNAs (miRNAs) are promising candidate biomarkers for diagnosis or prognosis for breast cancer. We investigated the prognostic role of exosomal miRNAs in serum samples derived from patients with breast cancer and compared miRNA expression between serum and tumor tissues.

**Methods:**

The miRNA profile derived from exosome between breast cancer patients with recurrence (n = 16) and without recurrence (n = 16) were compared by miRNA PCR array. Further, we examined the expression of miRNAs derived from tissues in the patients with breast cancer with (n = 35) and without recurrence (n = 39) by qRT-PCR.

**Results:**

Of 384 miRNAs, three miRNAs (miR-338-3p, miR-340-5p, and miR-124-3p) were significantly upregulated and eight (miR-29b-3p, miR-20b-5p, miR-17-5p, miR-130a-3p, miR-18a-5p, miR-195-5p, miR-486-5p, and miR-93-5p) were significantly downregulated in the patients with recurrence. We evaluated the expression of the miRNAs in tumor tissues. The patients with recurrence had higher levels of miR-340 at their primary site as well as in the serum. In contrast, miR-195-5p, miR-17-5p, miR-93-5p, and miR-130a-3p, derived from tumor tissues that were downregulated in the serum from patients with recurrence, were higher in the patients with recurrence than in those with no recurrence. In logistic regression analysis, miR-340-5p, miR-17-5p, miR-130a-3p, and miR-93-5p were significantly associated with recurrence.

**Conclusions:**

Several exosomal miRNAs may be useful biomarkers to predict breast cancer recurrence. We show the different expression patterns of miRNAs between tumor tissues and serum. These findings may suggest selective mechanism of release of exosomal miRNAs by cancer cells to regulate their progression.

## INTRODUCTION

In recent years, many new tools in the field of molecular profiling have been developed to accurately predict clinical outcome and response to therapy for early breast cancer [1]. In particular, we attempt to identify circulating cancer-specific biomarkers that are minimally invasive to analyze compared to tissue sampling. Several candidate biomarkers, including DNA, RNA, microRNA (miRNA), proteins and metabolites, have been proposed to date [2].

miRNAs are non-coding small RNA molecules with 19 to 25 nucleotides in length. miRNAs regulate gene expression at the post-transcriptional level by binding to 3’ or 5’ untranslated regions of target messenger RNAs (mRNAs) and leading to inhibition of translation or regulation of mRNA degradation [3, 4]. Besides their intracellular function, it is demonstrated that they play an important role in transmitting information to modulate their microenvironment. Recent studies indicate that miRNA may serve as oncogenes or tumor suppressor genes. During cancer development, their expression is frequently deregulated in several types of cancer, and abnormal expression levels are associated with cancer clinicopathological features and prognosis. Also in breast cancer, several miRNAs are reported to be valuable biomarkers for detection of cancer or metastases [5, 6]. Among them, miR-10, miR-215, miR-299-5p and miR-41 [7-9] have been suggested to be associated with metastatic breast cancer. In addition to the diagnostic and prognostic values, miRNA expression levels play a role in monitoring treatment response. For example, serum miR-125b is downregulated in HER2-overexpressing breast cancers and may be associated with chemoresistance in patients [10]. Similarly, miR-210 expression was reported to be associated with lower pathological complete response rates in patients who receive trastuzumab therapy [11]. Other putative miRNAs such as miR-200b-3p, miR-190a, and miR-512-5p involved in response to chemotherapy in breast cancer were suggested [12].

miRNAs are commonly incorporated in microvesicles or bound to lipoproteins such as HDL or associated with argonaute-2 (Ago2)-containing complexes in the blood [13]. It has been demonstrated that the majority of miRNAs in body fluids are concentrated in exosomes [14]. On the contrary, Arroyo et al. [13] found that most circulating miRNAs in plasma are cofractionated with Ago2. They also suggested that vesicle-associated versus Ago2 complex-associated miRNAs originate from different cell types.

Exosomes are small membranous vesicles (30–100 nm) including lipid, proteins, miRNAs and mRNAs, and are secreted from viable cells into the blood circulation [15]. They reflect the origin of the secreting cells, and “cargo” in exosomes may be important mediators of intercellular communication [16, 17]. Recently, exosomal miRNAs in body fluids have been used as one of the circulating biomarkers for the detection of several cancers [18-20], based on the findings indicating that cancer patients have elevated levels of tumor-derived exosomes in plasma or serum compared with those in healthy donors. In contrast to growing evidences about comprehensive circulating miRNAs, there is still limited information exclusively regarding exosomal miRNAs which appear to be more reflect to tumor content. Additionally, few reports about exosomal miRNAs evaluate their prognostic value in breast cancer.

In this study, we investigated the prognostic role of the exosomal miRNAs in serum samples derived from patients with primary breast cancer. We performed miRNA array analysis in 32 patients with and without recurrence, identifying several exosomal miRNAs significantly associated with recurrence. Further, we evaluated whether exosomal miRNAs in the serum reflect the origin of the primary tumor by comparing their expression levels between the serum and the tumor.

## RESULTS

### Comparison of exosomal miRNA profiles between patients with and without recurrence in breast cancer

Patients’ characteristics (n = 32) in the PCR array set are summarized in Table 1. The median follow-up period for patients without recurrence is 100 months. There is no significant difference in clinicopathological factors between patients with and without recurrence. We matched the tumor subgroup for both groups: 56 % had tumors of the luminal subtype, 25% had the HER2 subtype, and 19% had the triple negative (TN) subtype. Most patients received adjuvant or neoadjuvant treatment that included endocrine therapy, chemotherapy (anthracycline- and taxan-containing regimen) and trastuzumab therapy.

**Table 1:** Patient characteristics in the PCR array set

	Patients with no recurrence (n=16)	Patients with recurrence (n=16)	*P*
Mean age at diagnosis (min, max)	54 (30 - 79)	57 (43 - 75)	0.624
Menopausal status			
Premenopausal	6 (38%)	3 (19)	
Postmenopausal	10 (62%)	13 (81)	0.433
Tumor size (cm)			
Median ± SD	2.6 ± 0.9	2.3 ± 2.1	0.615
Nodal status			
Negative	8 (50%)	4 (25%)	
Positive	8 (50%)	12 (75%)	0.273
Clinical T			
T1	5 (31%)	5 (31%)	
T2	9 (56%)	8 (50%)	
T3	2 (13%)	0 (0%)	
T4	0 (0%)	3 (19%)	0.168
Stage			
1	1 (6%)	4 (25%)	
2	13 (81%)	9 (56%)	
3	2 (13%)	3 (19%)	0.256
Nuclear grade			
1,2	9 (56%)	10 (63%)	
3	7 (44%)	6 (38%)	1.000
Ki67 labeling index, median ± SD	49 ± 21.3	36 ± 26.2	0.396
ER status			
Negative	6 (38%)	7 (44%)	
Positive (≥1%)	10 (62%)	9 (56%)	1.000
PR status			
Negative	8 (50%)	8 (50%)	
Positive (≥1%)	8 (50%)	8 (50%)	1.000
Tumor subtype			
Luminal (ER and/or PR + and HER2 -)	9 (56%)	9 (56%)	
HER2 (ER and/or PR +/- and HER2 +)	4 (25%)	4 (25%)	
TN (ER and PR and HER2 -)	3 (19%)	3 (19%)	1.000
Adjuvant treatment			
Endocrine therapy	11 (69%)	9 (56%)	
Chemotherapy	7 (44%)	8 (50%)	
Trastuzumab	2 (13%)	3 (19%)	

ER, estrogen receptor; PR, progesterone receptor; HER2, human epidermal growth factor 2; SD, standard deviation; TN, triple negative.

**Figure 1:**
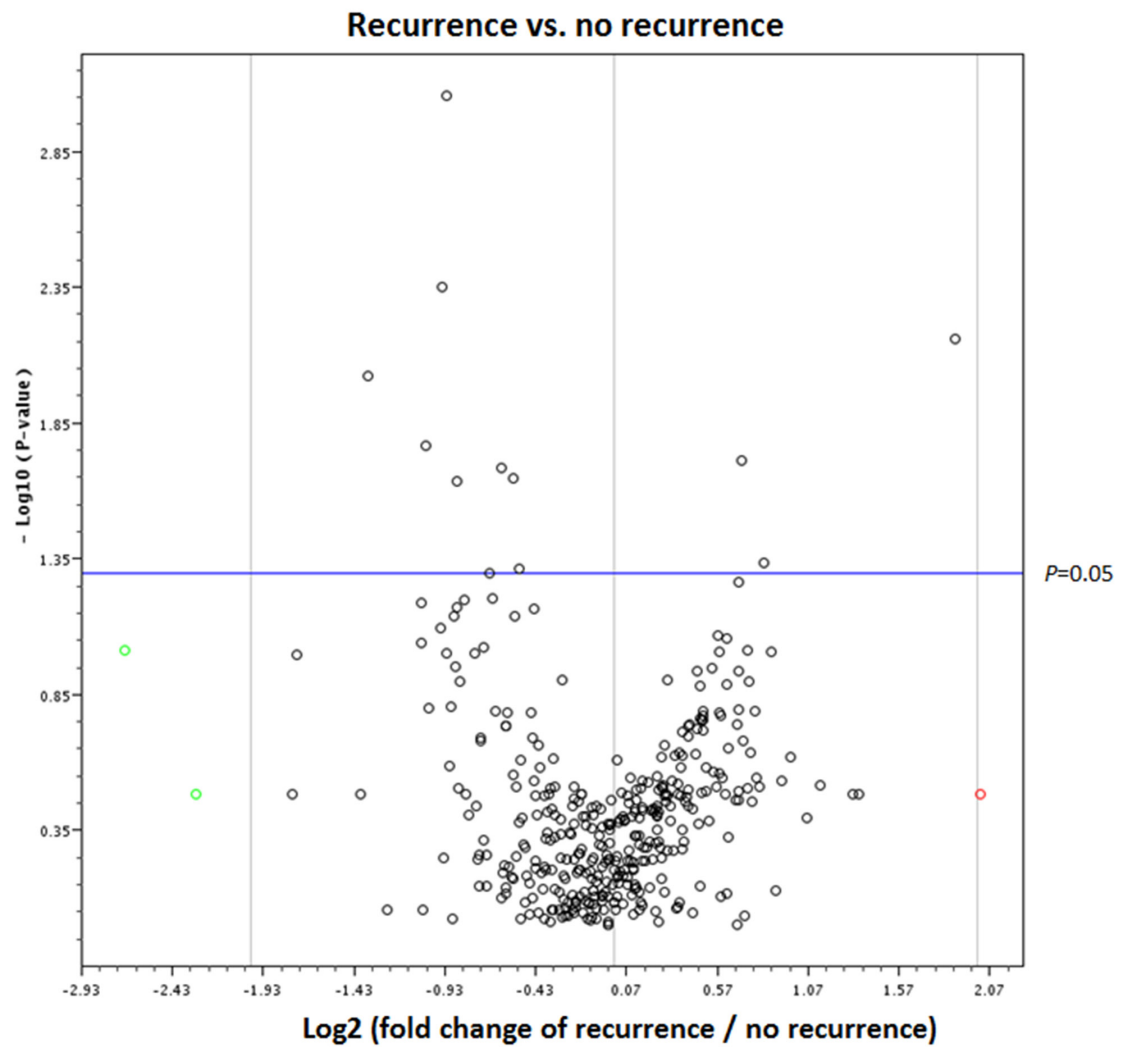
Volcano plot of PCR-based array between the patients with and without breast cancer recurrence The red circle indicates a fold change with values greater than 2, and green circles indicate a fold change with values lower than -2.

Analysis of PCR-based arrays for the expression of exosomal miRNAs was performed in serum samples at diagnosis to identify exosomal miRNAs related to recurrence. For all samples, we confirmed that at least 80% of the 384 miRNAs were expressed in the array set. Volcano plot of PCR array data show that 11 miRNAs were significantly expressed in the patients with recurrence (Figure 1).

Of the 384 miRNAs, three were expressed at significantly higher levels and eight were expressed at lower levels in the serum of the patients with recurrence compared with those without recurrence (Table 2); miR-338-3p, miR-340-5p and miR-124-3p were significantly upregulated, and miR-29b-3p, miR-20b-5p, miR-17-5p, miR-130a-3p, miR-18a-5p, miR-195-5p, miR-486-5p and miR-93-5p were significantly downregulated in the patients with recurrence. miR-124-3p had the highest fold change (3.67) among the upregulated genes and miR-93-5p (0.39) among the downregulated genes.

**Table 2:** Differentially expressed miRNAs related to breast cancer recurrence by PCR array

Mature ID	Fold change	*P*
Upregulation		
has-miR-338-3p	1.62	0.019
has-miR-340-5p	1.77	0.046
has-miR-124-3p	3.67	<0.01
Downregulation		
has-miR-29b-3p	0.69	0.049
has-miR-20b-5p	0.68	0.023
has-miR-17-5p	0.65	0.021
has-miR-130a-3p	0.55	0.023
has-miR-18a-5p	0.53	<0.01
has-miR-195-5p	0.52	<0.01
has-miR-486-5p	0.49	0.017
has-miR-93-5p	0.39	<0.01

### Analysis of miRNA expression in primary breast tumors

Cancer cells appear to secrete exosomal miRNA preferentially into the extracellular space, possibly to alter the cellular environment to favor tumor growth. We also compared the pattern of miRNA expression between the primary breast tumors with and without recurrence. We added more cases to the analysis, selecting 74 patients (35 with recurrence and 39 without recurrence), which included 21 patients used by the PCR array. Patients’ characteristics are shown in Supplementary Table 1. The patients with recurrence were more likely to have larger tumor size (*P* < 0.01) and advanced stage (P = 0.034), but there was no other difference in clinicopathological factors between the two groups.

**Figure 2:**
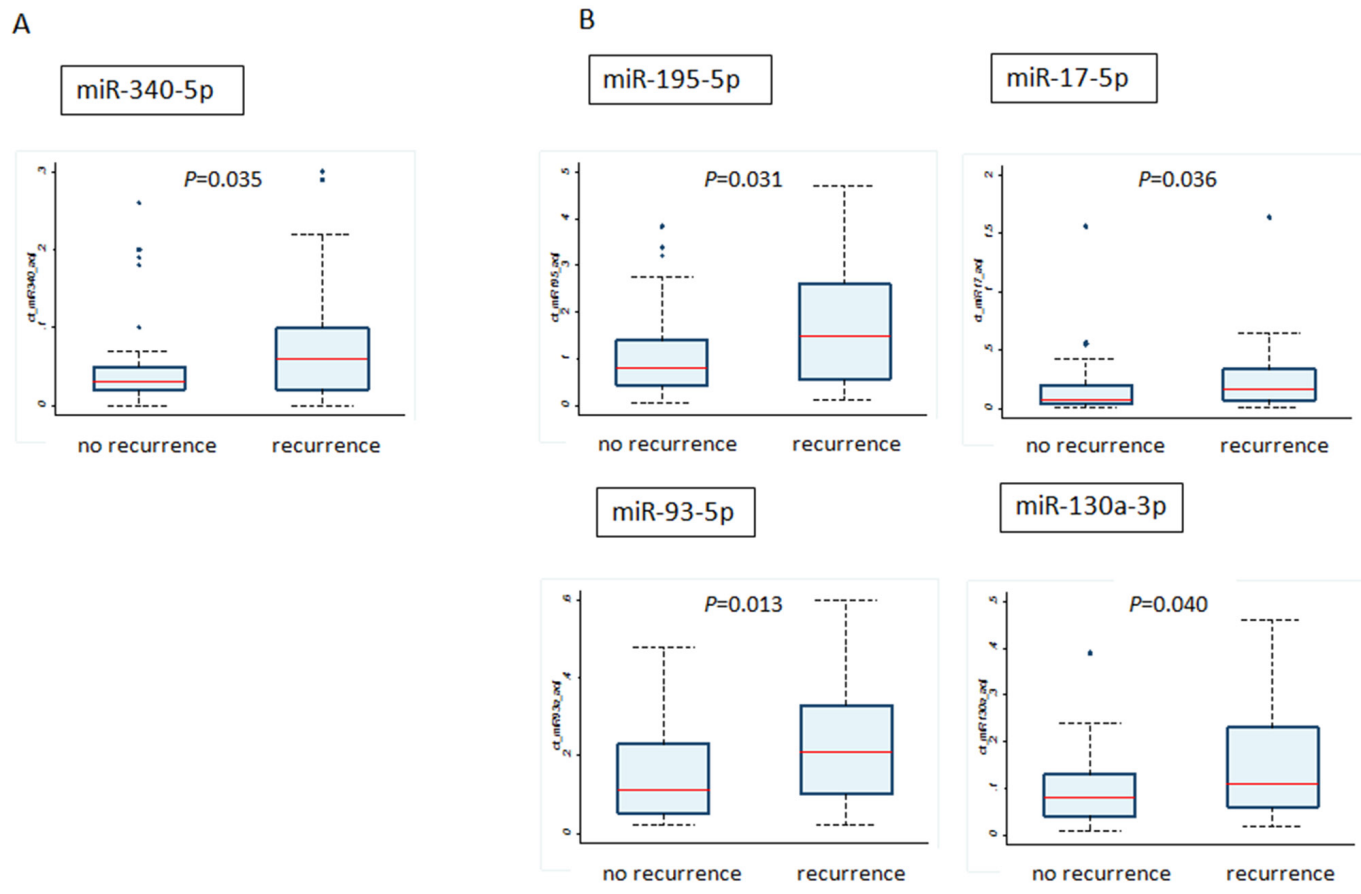
Comparison of miRNA levels between tumor tissues from patients with recurrence and without recurrence **(A)** The miRNA upregulated in the exosomes by PCR array; **(B)** the miRNAs downregulated in exosomes by PCR array.

Then we compared the expression of miRNAs in the tumors of patients with and without recurrence (Figure 2). Among the upregulated genes by PCR array in the serum, we observed the same tendency only for the miR-340-5p; the patients with recurrence have higher levels of miR-340 at their primary site as well as in the serum (Figure 2A). On the contrary, miR-195-5p, miR-17-5p, miR-93-5p and miR-130a-3p, which were downregulated in the serum of patients with recurrence, were higher in tumors of patients with recurrence compared with those without recurrence (Figure 2B). Regarding the remaining miRNAs, we found no statistical difference between the two groups. In addition, there were no significant correlations in miRNA expression between exosomes and breast tumors by Spearman’s correlation analysis (Supplementary Table 2). In univariate analysis, tumor size, miR-340-5p, miR-17-5p, miR-130a-3p and miR-93-5p were significantly associated with breast cancer recurrence (for each *P* < 0.05), and only miR-93-5p was also significant in multivariate analysis (*P* < 0.05) (Table 3).

**Table 3:** Logistic regression analyses for recurrence by miRNA expression in tissues

	Univariate		Multivariate	
miRNA (high vs. low)	OR (95% CI)	*P*	OR^a^ (95% CI)	*P*
has-miR-338-3p	1.97 (0.72–5.42)	0.189		
has-miR-340-5p	4.13 (1.38–12.4)	0.011	2.58 (0.69–9.66)	0.159
has-miR-124-3p	0.88 (0.31–2.47)	0.810		
has-miR-29b-3p	1.78 (0.62–5.10)	0.286		
has-miR-20b-5p	2.70 (0.93–7.85)	0.068		
has-miR-17-5p	3.25 (1.07–9.84)	0.037	1.61 (0.41–6.36)	0.500
has-miR-130a-3p	3.25 (1.07–9.84)	0.037	1.76 (0.46–6.69)	0.409
has-miR-18a-5p	1.69 (0.65–4.38)	0.282		
has-miR-195-5p	2.70 (0.93–7.85)	0.068		
has-miR-486-5p	1.16 (0.42–3.24)	0.777		
has-miR-93-5p	6.56 (1.91–22.5)	0.003	5.58 (1.46–21.3)	0.012

OR, odds ratio; CI, confidence interval.

aOR was adjusted for tumor size, miR-340-5p, miR-17-5p, miR-130a-3p, and miR-93-5p.

## DISCUSSION

A number of studies have shown that exosomal miRNAs derived from cancer cells are secreted into the circulation. Exosome-encapsulated miRNAs may represent ideal biomarkers for disease detection at an early stage. In the present study, we investigated the prognostic value of such exosome-derived miRNAs and demonstrated that 11 exosomal miRNAs at diagnosis were associated with breast cancer recurrence. Moreover, we show the different expression pattern of miRNAs between the tumor tissue and serum; miRNAs that were downregulated in the serum of patients with recurrence were highly expressed in the primary tumor compared with patients without recurrence. This finding is not fully consistent with the literature, but may suggest selective mechanism of release of exosomal miRNAs by cancer cells to regulate cancer progression.

Previous studies have mainly focused on miRNA signature based on their expressions in tissue specimens. In breast cancer, miRNAs have been demonstrated to be aberrantly expressed compared to normal breast tissues [21]. A number of miRNAs in breast tumors were identified as useful diagnostic and prognostic biomarkers [22] and have been shown to relate to various properties of cancer cells, such as proliferation [23], differentiation, angiogenesis [24], migration [25, 26], cancer stemness [27, 28] and epithelial to mesenchymal transition [29, 30].

There has also been great interest in circulating miRNAs as noninvasive biomarkers for cancer detection or progression. To date, the role of exosomal miRNAs as diagnostic and prognostic markers has been reported in ovarian [31], prostate [19], colon cancer [20] and melanoma [32]. In contrast, studies reporting the identification of exosomal miRNAs in breast cancer are limited, and most of them used breast cancer cell lines [33]. One recent study by Hannafon et al. [21] examined exosomal miRNAs using clinical samples and reported that the expression of plasma exosomal miR-1246 and miR-21 were significantly higher in the group of patients with breast cancer compared with the healthy population. Another report by Eichelser et al. [34] compared blood serum levels of circulating cell-free and exosomal miRNAs and reveled that exosomal miR-101 and miR-373 were significantly different between patients with breast cancer and benign tumors.

Our comprehensive analysis of gene expression showed that 11 miRNAs were significantly aberrantly expressed in the group with recurrence (Table 2). These miRNAs, except for miR-17-5p, miR-20b-5p, miR-93-5p and miR-18a-5p, are considered to be tumor suppressors in various types of cancer according to the literature [35-42]. Some have the evidence on the biological function within cancer cells, but others have no or controversial results; the biological roles of miR-338-3p, miR-29b-3p, miR-486-5p, and miR-130a-3p has not been clarified yet in breast cancer. Identification of novel miRNAs may become a key step in the future development of exosome biomarkers for breast cancer.

Moreover, we compared miRNA expressions between exosomes and tumor tissues at diagnosis. Some studies reported that the miRNA content of the originating cancer cells is similar to that found in circulating exosomes [31]. In the present study, however, miRNA expression in exosomes did not mirror that in the originating tumor tissues (Figure 2 and Supplementary Table 2). Among the miRNAs expressed in primary tumor tissues, miR-340-5p, miR-17-5p, miR-130a-3p and miR-93-5p derived from breast cancer patients with recurrence were highly expressed compared with those from patients without recurrence. However, those miRNAs, except for miR-340-5p, were downregulated in the exosomes of patients with recurrence. miR-17-5p belongs to the miR-17-92 cluster, which is involved in tumor proliferation by controlling the PI3K/Akt/mTOR pathway [43]. Jin et al. showed that miR-17-5p was overexpressed in TN breast cancer (TNBC) and could inhibit ribosomal translation of tumor suppressor genes [44]. Other reports have also shown that miR-93-5p was upregulated in cervical carcinoma or the expression levels of miR-93-5p in TNBC tissues is significantly higher than in non-TNBC tissues [37]. Regarding miR-340-5p, the patients with recurrence had higher levels of miR-340 at their primary site as well as in the serum. Several studies have suggested the role of miR-340 as tumor suppressor gene [45, 46], but one report demonstrated the association between miR-340 and chemoresistance [47]. Raychaudhuri et al. reported that the patients with high expression of miR-340 in pretherapeutic biopsies in breast cancer were less likely to achieve pathological complete response (pCR). MiR-340 is predicted to regulate several genes which are associated with tumor growth and proliferation including Jun- and Fos oncogene, and Cyclin Dependent Kinase 5 [47].

A few reports have demonstrated the mechanism of secretion of miRNAs, and secretory miRNAs seem to play a pivotal role as signaling molecules in cancer. However, the mechanisms controlling the secretion of miRNAs into exosomes or the retention of miRNAs inside tumor cells still remain unknown. Pu et al. [48] investigated tissue-specific and plasma miRNA profiles in non-small cell lung cancer and found that the expression levels of specific miRNAs were different between tissues and plasma. Similarly, Matamala et al. [49] have compared miRNA expression levels in breast cancer patients and healthy individuals, leading to the identification of five differentially expressed miRNAs in the plasma. However, some of the miRNAs analyzed were deregulated in opposite directions compared with tumors. According to previous studies, tumors themselves release miRNAs into the circulation [50]. There may be some difference in miRNA profiles within the cancer tissue, which require to grow at the primary site, and those in the exosomes released in the serum, which modulate the extracellular environment. Moreover, exosomes in serum can originate from not only cancer cells but also from nonmalignant cells such as circulating blood cells, although this implication is considered to be substantially low. It is, therefore, possible that the alteration in miRNA expression seen in serum reflects the comprehensive feature of systemic response, displaying difference in exosomal and tissue miRNA profiles.

Our research has several advantages on the study of exosomal miRNAs. Measuring of miRNAs using serum exosomes is ideal in terms of miRNA integrity. Encapsulation by exosomes can protect miRNAs in extreme conditions in the serum, thus maintaining miRNA quality. In our study, we used ExoQuick reagent for exosome isolation while the commonly used methods to isolate exosome is ultracentrifugation. We showed successful isolation of exosomes and miRNA analysis. Compared to ultracentrifugation, commercially available kits like ExoQuick are less time consuming and need lower volume of patient serum [2].

In contrast, several limitations exist in our study. First, there is no reliable endogenous gene for normalization of exosomal miRNAs. We selected miR-16-5p, miR-423-3p and miR-191-5p as reference genes, because they were constantly expressed through our data and were recommended as reference miRNAs in the literature [34, 51]. Second, the stability of exosome in stored patient sample for long term period is unclear. However, Kalra et al. [52] assessed the stability of exosome in plasma and showed that exosome stored at -80 °C were highly stable compared to other storage conditions. We also stored serum sample at -80 °C, but need further research to assure the stability of exosome or miRNAs. Third, the technology functions by ExoQuick cannot avoid mixing of non-exosomal contents with similar size such as apoptotic debris and blood borne cell-derived exosomes. This may cause the difference in the miRNA expression pattern between exosomes and tumor tissues. Finally, we combined all tumor subtypes in our study because we didn’t have many samples available. However, the prognostic significance of each miRNA may differ among tumor subtype. We should perform stratified analyses by tumor subtype to increase the performance for prediction of recurrence in breast cancer.

In conclusion, here we show that 11 exosomal miRNAs are associated with breast cancer recurrence and may be potential candidate biomarkers for breast cancer prognosis. In contrast to growing evidence of the clinical use of circulating miRNAs as diagnostic tools, there is limited data regarding their prognostic impact. Accordingly, we provide additional evidence on it. Interestingly, we report also different miRNA expression patterns between tumors and exosomes, which can be used as prognostic factors. Further clinical and functional studies focusing on these identified miRNAs are needed to validate our findings.

## MATERIALS AND METHODS

### Study population

We selected the patients with primary breast cancer with recurrence (n = 16) and without recurrence (n = 16) for analysis of miRNA PCR array. All patients received surgery and treatment at Kumamoto University Hospital between 2003 and 2011 and were diagnosed with invasive breast carcinoma. We matched the tumor subtype for both group as mentioned in the Results section. Tumor subtypes were defined according to the expression of ER, PR and HER2. ER and PR were considered positive if more than 1% of nuclei were stained. HER2 expression was determined by IHC staining based on the Hercep test. Ki67 was scored as the percentage of nuclear-stained cells out of all cancer cells in the hot spot of the tumor, regardless of the intensity in a ×400 high-power field (Ki67 labeling index).

Neoadjuvant or adjuvant treatment was assigned to each patient according to their risk on the basis of clinical parameters, and in accordance with the recommendation of theSt. Gallen International Expert Consensus on primary therapy of early breast cancer at the time. All serum samples (500 µL) were collected from breast cancer patients before surgery and treatment.

For analysis of tumor miRNAs, we selected additional breast cancer patients with (n = 35) and without recurrence (n = 39), including 21 patients used for the PCR array analysis. All tumor tissues were obtained from patients before surgery or treatment.

Written informed consent was obtained from all subjects for the collection and research use of breast tumors. Our complete study was approved by the ethics committee of Kumamoto University Graduate School of Medical Sciences.

### Exosome isolation and RNA extraction

We store the serum sample at – 80 °C before use in all patients. Exosomes in serum samples (500 µL) were extracted using ExoQuick (System Biosciences, Palo Alto, CA, USA) according to the manufacturer’s instructions. In brief, we combined the reagent into the serum sample and incubated at 4°C for at least 30 min to precipitate the exosome pellet. The size of exosomes was confirmed using the Nanosight LM10 instrument (Nanosight, Tokyo, Japan, Supplementary Figure 1A) and the exosomal membrane marker (CD63) was confirmed by Western blot analysis (Supplementary Figure 1B). Thereafter, total RNAs that include small RNA fraction from exosome pellet were isolated using SeraMir™ Exosome RNA Amplification Kit (System Biosciences) according to the manufacturer’s instructions. The quality of RNA samples and small RNA fraction was checked via the Agilent 2100 Bioanalyzer (Agilent Technologies, Supplementary Figure 1C). The RNA samples were immediately stored at -80°C until they were used.

### Western blotting

The pelleted exosomes extracted using ExoQuick (System Biosciences) were resuspended in phosphate-buffered saline following the protein analysis. The proteins were loaded on Mini-PROTEAN^®^ TGX™ gel (Bio-Rad Laboratories, Hercules, CA, USA) and electrophoretically transferred onto Trans-Blot^®^ Turbo™ Mini Nitrocellulose membranes (Bio-Rad Laboratories). The membranes were blocked and incubated overnight at 4°C with the primary antibody anti-CD63 (1:200; Santa Cruz Biotechnology, Fremont, CA, USA). Proteins were visualized with horseradish peroxidase-conjugated secondary antibodies (anti-rabbit IgG, HRP-linked antibody; Cell Signaling Technology, Tokyo, Japan) followed by chemiluminescence detection (Pierce Western Blotting Substrate Plus; Thermo Scientific, Tokyo Japan).

### Evaluation of miRNA PCR array in serum

Total RNA (125 ng) extracted from exosomes was reverse transcribed to cDNA using miScript II RT kit (Qiagen, Valencia, CA, USA), according to the manufacturer’s protocol. Then, exosomal miRNA profiling was performed using miRNA PCR array (Qiagen) containing primers for 384 human mature miRNAs according to the manufacturer’s protocol. For the normalization of real-time PCR, we selected miR-16-5p, miR-423-3p and miR-191-5p, because they were constantly expressed in our data and recommended as reference miRNAs in the literatures [34, 51].

### Evaluation of miRNA expression in tumor tissues

Total RNA from tissue samples was isolated using the AllPrep^®^ DNA/RNA/miRNA Universal kit (Qiagen) according to the manufacturer’s protocol. RNA was quantified by measuring the A260/A280 absorbance ratios (Nano-Drop Technologies, Wilmington, DE). Total RNA (0.5 μg) was reverse transcribed to cDNA using miScript II RT kit (Qiagen), according to the manufacturer’s protocol. Each real-time quantitative PCR was performed with 0.03 μL of the cDNA, 10×miScript Universal Primer, 10×miScript Primer Assay and 2×QuantiTect SYBR Green PCR Master Kit in the ABI Prism 7500 (Applied Biosystems, Carlsbad, CA). Each reaction (10 μL samples) was performed under the following conditions: initialization for 15 min at 95 °C, and then 40 cycles of amplification, with 15 s at 94°C for denaturation, 30 s at 55°C for annealing, and 30 s at 70°C for elongation. The expression of target genes was normalized against RNU6B and miR-16-5p. The relative expression of each miRNA was calculated using the 2^-∆∆Ct^ method.

### Statistical analysis

In PCR array analysis, candidate miRNAs with differential expression between the patients with and without breast cancer recurrence were determined based on Student’s t-test. Significant miRNAs were defined with p-value < 0.05 and at least 1.5 fold change. The significance of differences in categorized demographic variables was evaluated using Chi-square or Fisher’s exact test and the nonparametric Mann-Whitney *U* test. The correlation between exosomal and tissue miRNA levels was analyzed using Spearman’s correlation coefficient. Logistic regression methods were adopted for univariate and multivariate analyses to assess the associations of each miRNA with breast cancer recurrence. Odds ratios (ORs) and 95% confidence intervals (CIs) were calculated. All statistical analyses were carried out using STATA ver.12 (Stata Corp, College Station, TX). All tests were two-sided and p-values < 0.05 were considered statistically significant.

## SUPPLEMENTARY MATERIALS FIGURE AND TABLES



## Abbreviations

Ago2: argonaute-2
ER: estrogen receptor
HER2: human epidermal growth factor receptor 2
MiRNA: microRNA
MRNA: messenger RNA
OR: odds ratio
PR: progesterone receptor
TN: triple negative
