# Supplementary material for: Differential expression of exosomal miRNAs between breast cancer patients with and without recurrence
Source: Oncotarget. 2017 Jul 22;8(41):69934–44. doi: 10.18632/oncotarget.19482 (PMC5642528; doi:10.18632/oncotarget.19482)
Supplement: Supplementary file 1 [file oncotarget-08-69934-s001.pdf]

Differential expression of exosomal miRNAs between breast cancer patients with and without recurrence

SUPPLEMENTARY MATERIALS

Supplementary Table 1: Patients' characteristics for tissue miRNA analyses

	Patients without recurrence (n = 39)	Patients with recurrence (n = 35)	*P*
Mean age at diagnosis (min, max)	61 (30–81)	57 (30–91)	0.171
Menopausal status			
Premenopausal	11 (28%)	12 (34%)	
Postmenopausal	28 (72%)	23 (66%)	0.573
Tumor size (cm)			
Median ± SD	1.8 ± 1.02	3.0 ± 2.55	<0.01
Nodal status			
Negative	23 (59%)	16 (46%)	
Positive	16 (41%)	19 (54%)	0.254
Stage			
1	15 (38%)	8 (23%)	
2	22 (56%)	18 (51%)	
3	2 (5%)	9 (26%)	0.034
Nuclear grade			
1,2	28 (72%)	23 (66%)	
3	11 (28%)	12 (34%)	0.573
Ki67 labeling index, Median ± SD	33 ± 22.1	33 ± 26.5	0.691
Tumor subtype			
Luminal (ER and/or PR+ and HER2-)	29 (74%)	22 (63%)	
HER2 (ER and/or PR+/- and HER2+)	7 (18%)	5 (14%)	
TN (ER and PR and HER2-)	3 (8%)	8 (123%)	0.206
Adjuvant treatment			
Endocrine therapy	30 (77%)	20 (57%)	
Chemotherapy	9 (23%)	19 (54%)	
Trastuzumab	4 (10%)	2 (6%)	

ER, estrogen receptor; PR, progesterone receptor; HER2, human epidermal growth factor 2.

Supplementary Table 2: Correlation between miRNA expression in exosomes and tissues

miRNA	Spearman's correlation coefficient	*P*
has-miR-338-3p	-0.075	0.76
has-miR-340-5p	-0.233	0.34
has-miR-124-3p	-0.153	0.51
has-miR-29b-3p	-0.045	0.85
has-miR-20b-5p	-0.128	0.61
has-miR-17-5p	-0.166	0.48
has-miR-130a-3p	-0.396	0.084
has-miR-18a-5p	-0.298	0.23
has-miR-195-5p	-0.336	0.14
has-miR-486-5p	-0.126	0.6
has-miR-93-5p	-0.434	0.063

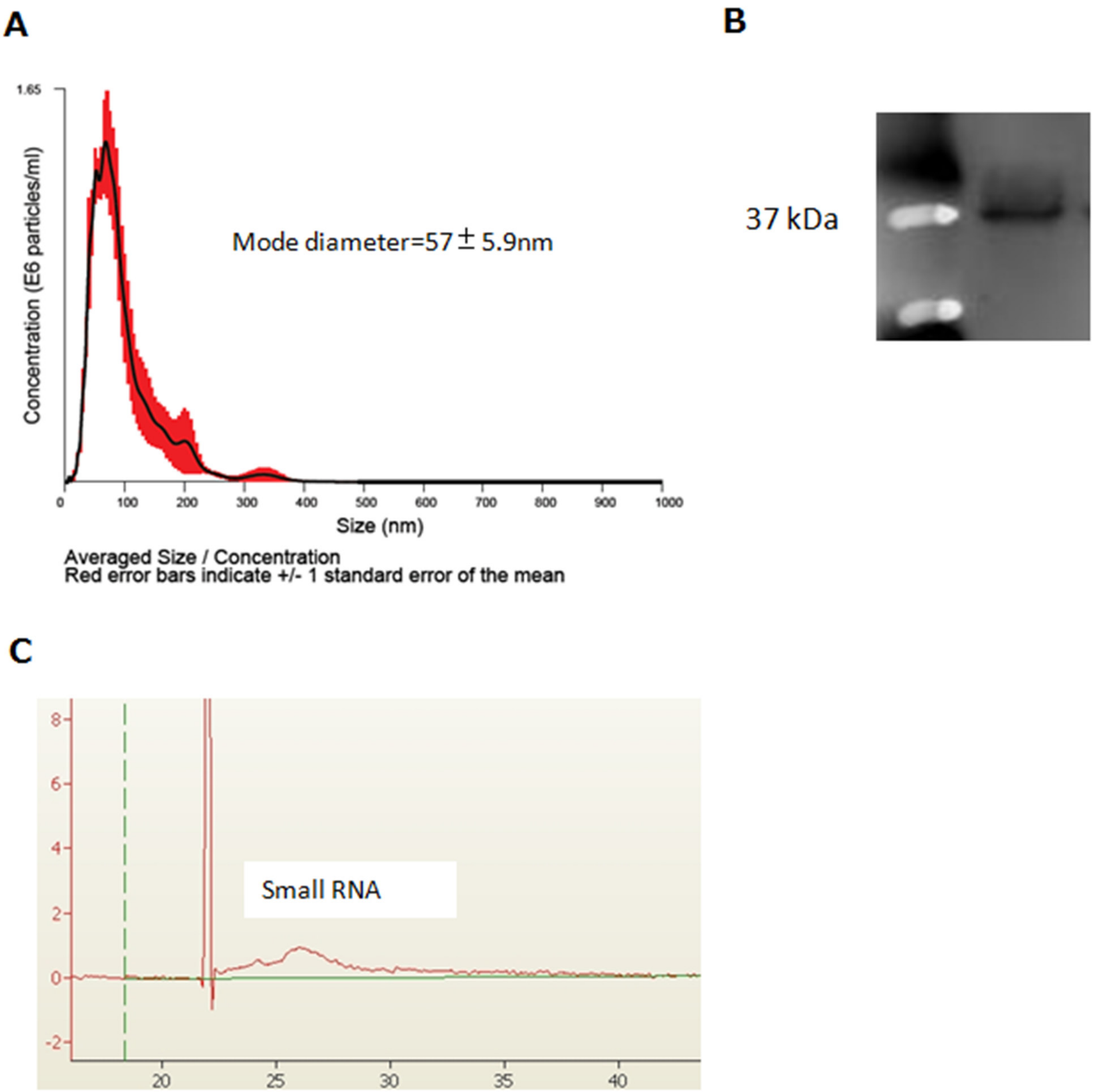

Supplementary Figure 1: (A) Image of the size of exosomes using the Nanosight LM10 instrument. The most frequently found size is 57 nm. **(B)** Western blot analysis of the exosomal membrane marker CD63. **(C)** The quality of RNA samples and small RNA fraction by the Agilent 2100 Bioanalyzer.
